# Postural Assessment Scale for Stroke Patients in Acute, Subacute and Chronic Stage: A Construct Validity Study

**DOI:** 10.3390/diagnostics11020365

**Published:** 2021-02-21

**Authors:** Cecilia Estrada-Barranco, Roberto Cano-de-la-Cuerda, Vanesa Abuín-Porras, Francisco Molina-Rueda

**Affiliations:** 1 Department of Physiotherapy, Faculty of Sport Sciences, Universidad Europea de Madrid, Villaviciosa de Odón, 28670 Madrid, Spain; CECILIA.ESTRADA@universidadeuropea.es (C.E.-B.); VANESA.ABUIN@universidadeuropea.es (V.A.-P.); 2 Physical Therapy, Occupational Therapy, Rehabilitation and Physical Medicine Department, Faculty of Health Sciences, Universidad Rey Juan Carlos, 28032 Madrid, Spain

**Keywords:** functional assessment, gait, outcome measures, postural assessment scale for stroke patients, postural balance, stroke

## Abstract

(1) Background: Observational scales are the most common methodology used to assess postural control and balance in people with stroke. The aim of this paper was to analyse the construct validity of the Postural Assessment Scale for Stroke Patients (PASS) scale in post-stroke patients in the acute, subacute, and chronic stroke phases. (2) Methods: Sixty-one post-stroke participants were enrolled. To analyze the construct validity of the PASS, the following scales were used: the Functional Ambulatory Category (FAC), the Wisconsin Gait Scale (WGS), the Barthel Index (BI) and the Functional Independence Measure (FIM). (3) Results: The construct validity of the PASS scale in patients with stroke at acute phase was moderate with the FAC (*r* = −0.791), WGS (*r* = −0.646) and FIM (*r* = −0.678) and excellent with the BI (*r* = 0.801). At subacute stage, the construct validity of the PASS scale was excellent with the FAC (*r* = 0.897), WGS (*r* = −0.847), FIM (*r* = −0.810) and BI (*r* = −0.888). At 6 and 12 months, the construct validity of the PASS with the FAC, WGS, FIM and BI was also excellent. (4) Conclusions: The PASS scale is a valid instrument to assess balance in post-stroke individuals especially, in the subacute and chronic phases (at 6 and 12 months).

## 1. Introduction

The balance evaluation is a priority objective in the stroke rehabilitation process. Balance while sitting in the acute stage has been related to shorter hospital rehabilitation stays [1] and better levels of functionality. Most daily-life activities required that the subject could maintain a stable sitting position for their performance as a prerequisite [2]. In addition, it is considered an important predictive factor concerning walking ability to achieve a stable gait within 6 months after stroke. Some authors point out the importance of testing walking ability in initial assessments to help clinical decisions [3].

Proximal stability and balance are a prerequisite for gait [4,5,6,7]. Thus, early intervention in the acute stage in patients with stroke can improve the evolution of gait in the following stages [8] and recovery from activities of daily living [9]. In this sense, different studies have shown that therapeutic approaches aimed at improving standing balance and compensatory strategies are more important than recovery of strength for improving the functionality and, specifically, gait function in stroke patients [10,11].

The most widely used scales to assess balance in stroke patients are the Berg Balance Scale (BBS) and the Postural Assessment Scale for Stroke Patients (PASS) [12]. Both have shown a high correlation between their scores [13,14], although the PASS scale is the only one originally designed to evaluate stroke patients.

The PASS scale evaluates balance with the patient lying, while sitting and standing. It involves 12 items which score can vary from 0 to 3, with 0 being the lowest level of functionality and 3 the highest; the total score can be 36 [15]. It was developed from the relationship between the ability to maintain a posture and to ensure balance while changing position. The PASS scale has been shown to be more sensitive to changes than the BBS in patients with more severe stroke in the initial stages [13]. It has also been shown to be useful to measure the progress of patients with Pusher Syndrome [16].

Regarding the psychometric properties of the PASS scale, the construct validity has been studied with respect to the Barthel Index (BI), showing a high correlation index [13]. Furthermore, it has been shown to have an excellent correlation with other balance scales [13,14]. However, until the present work, its correlation with scales that evaluate walking has not been studied. Balance in acute stages of stroke has a predictive value on gait recovery [3]. Both objectives are a priority in the rehabilitation of stroke patient. In addition, its construct validity has not been analysed with the Functional Independence Measure (FIM), which has shown a greater capacity to detect changes in functionality than the BI.

The objective of this work is to analyse the construct validity of the PASS scale in stroke patients at 8 weeks, 3 months, 6 months, and 12 months after stroke.

## 2. Materials and Methods

### 2.1. Design

An observational study was conducted following the STROBE (Strengthening the Reporting of Observational studies in Epidemiology) guidelines [17]. A retrospective secondary analysis was performed using data from a Spanish hospital specializing in stroke patients. Patients are usually admitted in an early stage (acute phase) after stroke. The study was approved on 30th November 2016 by the Local Ethics Committee (031020168316).

### 2.2. Participants

One researcher (C.E.-B.) performed the data compilation. This information was extracted from the clinical assessment documented in the patient’s files. The screening eligibility was conducted from October 2016 to May 2017. Inclusion criteria were: (1) Patients whose treatment included as a SMART objective gait and balance therapy programs [18]; (2) Acute stage (less than 8 weeks from a stroke); (3) Stroke diagnostic confirmation performed through resonance imaging or computed axial tomography; and (4) Patients that followed the same standardized gait and balance therapy program. Exclusion criteria were: (1) Unbalanced cardiovascular status; (2) Additional relevant musculoskeletal or neurological conditions and (3) incapability to follow instructions. Demographic characteristics were also compiled.

### 2.3. Procedure

To measure the construct validity of the PASS in different stages of the post-stroke process (acute, subacute and chronic) the following scales were used: the Functional Ambulatory Category (FAC), the Wisconsin Gait Scale (WGS), the Barthel Index (BI) and the Functional Independence Measure (FIM). The data compiled consisted in the information from 4 assessment sessions that were conducted by the same clinician in specific moments after stroke: at the time of admission (acute stage), after three months (subacute stage), an finally after 6 and 12 months (chronic stage) [19]. Table 1 shows the items evaluated by the PASS scale.

In 1984, Holden et al. defined a functional walking test, the FAC. It is designed for the assessment of gait capability. This test stablishes six levels of ambulation ability regarding the quantity of physical aid needed during gait. The FAC is validated as an evaluation tool in stroke patients. Moreover, its reliability and responsiveness had been demonstrated [20]. The test is considered to address the Activity domain of the International Classification Function, and its measure domain, Motor.

Concerning the WSG, the aim of the scale is to evaluate gait in post-stroke patients. The WSG assess walking ability components through 14 items. Thirteen items in the scale target the behavior of the lower limb during the gait cycle, and the last one focuses in handheld gait aids. The score frame varies from 1 (labelled as “normal”) to 3 (labelled as “atypical”). An exception to this statement would be the first item (1 to 5) and the 11th (1 to 4) [21,22]. The highest scoring would be 13.35, and the lowest 42. Thus, elevated numbers would translate clinically into poor gait ability, being the minimal clinically significant difference established in 2.25 points. 

Another test used for the analysis was the BI. This instrument evaluates the capacity of the patient for the performing of ten basic activities in daily life, obtaining a quantitative approximation of the subject’s level of dependency. The scoring frame varies from 0 (totally dependent) to 100 (totally independent). The BI shows high reliability, validity, and change detection [23]. It was one of the first attempts to quantify disability, and it is still one of the most useful tools for clinicians when assessing dependence.

Finally, the FIM evaluates physical, psychological, and social function through 18 items. Scoring range varies from the lowest level (1) that would imply complete dependence and the highest (7), which would mean total independence with no helper [24]. The tasks assessed in the test include self-care activities, bowel and bladder control, transfers, locomotion, social cognition, and communication.

Each one of the four tests has proved to be valid and reliable for post-stroke patients [25,26,27,28,29].

### 2.4. Sample Size

In order to calculate sample size, G*Power software (G*Power v. 3.1.9.2; Statistical Power Analyses Inc., L.A.; USA) was used, with a two-tailed hypothesis, a *p*-value of 0.05 [30] and a power of 0.95 [31]. The resultant sample size was 46 subjects.

### 2.5. Statistical Analysis

Statistical analysis was performed using SPSS statistical software (v. 20.0). To assess the normality of the distribution of the variables, Shapiro–Wilk and Kolmogórov–Smirnov tests were performed, and the results showed a non-normal distribution. To explore the construct validity, the Spearman correlation coefficient with 95% Confidence Interval (CI) was used. Correlation coefficients (CC) of 0.00 to 0.49 were interpreted as poor, those of 0.50 to 0.79 as moderate, and those of 0.8 or higher as excellent [32].

The authors considered the overlapping of the CI to establish that there was a difference in the CC amongst the various times of assessment. Therefore, following a previous study from Knol et al. [10] and the CI adjustment was performed with an 83.4% confidence level (formula 11) to reach an alpha error probability of 5%.

Bonferroni correction was conducted to adjust the multi-test probability addition. With four comparisons, a *p*-value of α = 0.05/4 (0.0125) was considered statistically significant. 

## 3. Results

**Figure 1:**
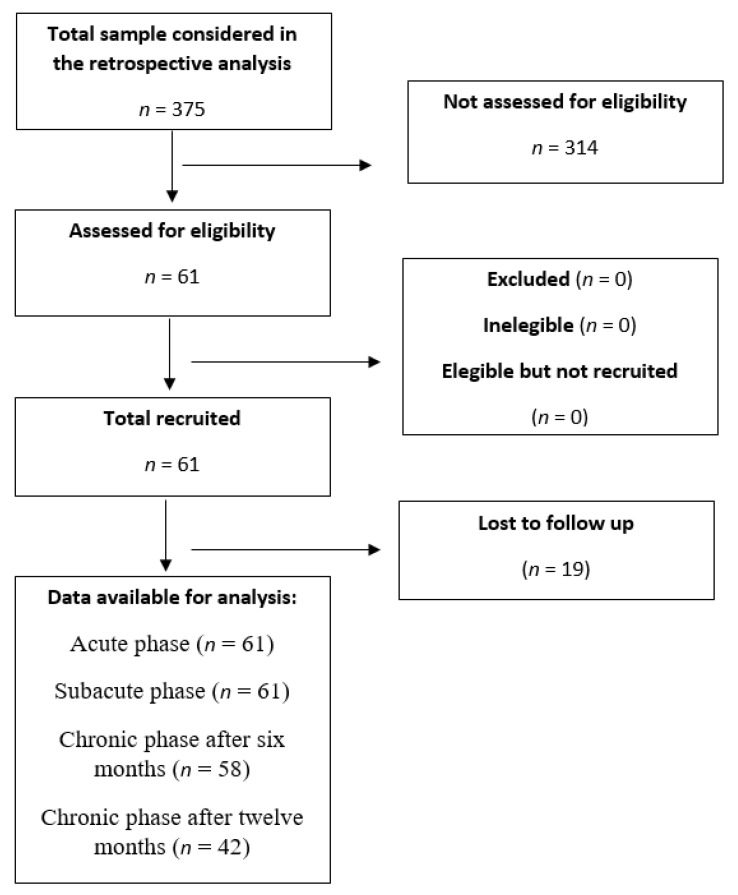
Strobe flow chart.

Data from 375 post-stroke patients of the hospital were retrospectively analyzed for eligibility. Finally, data from 61 subjects were collected (Figure 1). Characteristics of the study sample are shown in Table 2.

From the initial 61 subjects assessed during their acute stage (17.07 ± 13.21 days), 61 patients remained in the hospital during the subacute stage (107.06 ± 13.25 days), 58 in the chronic stage after six months (197.08 ± 13.25 days) and 42 patients after 12 months (382.06 ± 9.8 days; Figure 1), derived from the same individuals, which is also considered chronic stage, which is also considered chronic stage. The principal reason for subject loss in the study was discharge from the facility.

From the compiled data of the sample, 40 male and 21 female patients were retrospectively enrolled. The mean age of the subjects was 62.75 ± 13.31 years. Forty-seven had an ischemic stroke, and 14 had a hemorrhagic stroke. Concerning the affected hemisphere, 34 had right affectation, 21 left, 3 bilateral, and 3 subcortical affectation. The main vascular territory affected in the sample was the middle cerebral artery (38 subjects), followed by diffuse affectation (10), basilar (8) anterior (1) and internal carotid artery (1). Finally, the National institute of Health Stroke Scale score mean after admission was 13.48 ± 6.185.

Table 3 shows the mean of the timeframe since the stroke event and outcome measure scores at the four assessment points (acute, subacute, and chronic phase). The mean values show that the time effect was positive for all outcome measure scores.

The construct validity of the PASS scale in post-stroke patients at acute phase was moderate with the FAC (*r* = −0.791), WGS (*r* = −0.646) and FIM (*r* = −0.678) and was excellent with the IB (*r* = 0.801).

At subacute phase, the construct validity of the PASS scale was excellent with the FAC (*r* = 0.897), WGS (*r* = −0.847), FIM (*r* = −0.810) and IB (*r* = −0.888). So, all correlations values improved at chronic stages. At six and 12 months, the construct validity of the PASS with the FAC, WGS, FIM and BI was also excellent (Table 4).

## 4. Discussion

The aim of this research was to explore the construct validity of the PASS scale in post-stroke patients in the acute, subacute, and chronic stages after stroke. To the author’s knowledge, no construct validity studies of the PASS scale with observational gait scales, with independence scales for gait, or with the FIM scale have been published up to this date. Moreover, the patients were assessed at four time points after stroke to determine the ability of the PASS scale to measure the evolution of balance in stroke patients.

The results showed a moderate correlation between the PASS scale and the FAC, WGS and FIM for patients with stroke in the acute phase and excellent with the BI in this same stage. On the other hand, the correlation was excellent in the subacute and the chronic stages (6 and 12 months) for all the scales studied.

The main contribution of the PASS scale compared to other similar scales is the assessment of balance in different positions, establishing a relationship between postural control and balance. For this, it is possible to detect changes in the acute stage that other scales cannot detect due to deficits such as unilateral neglect and altered subjective postural vertical alignment in those patients who have not yet achieved standing [32,33,34]. This could explain the moderate correlation in the acute stage with the other scales, since in this period there is greater heterogeneity in terms of functional status. For example, patients who have good standing balance do not have independent walking ability. However, as neurological damage stabilizes and they receive rehabilitation, their functionality improves. However, the PASS scale can be useful in this acute period precisely because it can evaluate individuals with very poor functional levels. The results of this work confirm the validity of the PASS scale in stroke patients, mainly in the subacute and chronic phase (6 and 12 months).

Regarding functionality, the results published by Mao et al. [13] on the construct validity of the PASS with the BI are also confirmed. Construct validity with the FIM has not so far been studied. The FIM scale has shown adequate sensitivity in general, especially regarding the burden of care required by the patient [35]. Specifically, it has been proven to be robust, in terms of its psychometric properties, in hospitalized stroke patients [36]. Our results showed an excellent correlation in the subacute and chronic phases between the FIM and the PASS scale.

Regarding gait independence and gait characteristics, the correlation between the PASS scale and FAC, as well as with WGS, was excellent in patients in subacute and chronic stages (at 6 and 12 months). The FAC assesses the patient’s ability or inability to walk independently, and the WGS assesses the characteristics of the gait pattern [28,29]. Moreover, the PASS SCALE has been proved as a valid tool for predictive purposes, being reliable as a prediction for gait ability in the subacute stage [37]. The high correlation with the PASS scale demonstrates the relationship between the components necessary in balance to achieve independence in walking. In addition, the improvement of isolated gait components is not necessarily related to an increase in global functionality. Nevertheless, PASS scale has demonstrated a high correlation with WSG, which measures different aspects of gait pattern [38].

The PASS scale has demonstrated an excellent correlation with other balance scales; however, its correlation with other constructs, such as gait, has not been studied to date. The relationship between balance, gait, and functionality has been studied with other scales such as the BBS [39]. Also, given the enormous variability of stroke patients, measuring the psychometric properties in different periods after the stroke would allow us to choose the most appropriate instruments for evaluating patients.

Some limitations must be admitted in this study. As the nature of the investigation is retrospective, the conclusions of the research could have been altered by the fact that some of the subjects may not have been included in the analysis. Type or severity of the stroke’s manifestations was not taken into consideration. Larger samples would be needed to assess this latest fact, as the outcomes could be affected by both the type of stroke [ischemic or hemorrhagic] or level of severity (mild, moderate or severe) [40], regarding the psychometric characteristics of the measures. Furthermore, our study shows the construct validity of the PASS scale with other scales related to gait, and functionality scales, in stroke patients during a period of time in a rehabilitation context, so results are inferred to the inpatient rehabilitation context. Nevertheless, it is perceptible that the procedures used in this research to evaluate ambulation and functionality variables needed quite simple equipment accessible to any rehabilitation clinic and a suitable amount of time of analysis.

## 5. Conclusions

Our results show that the PASS scale is a useful instrument to assess balance in stroke patients. The PASS presents an adequate construct validity with gait and functional scales. The correlation between the scales was moderate-excellent especially, in the subacute and chronic phases.

## Figures and Tables

**Table 1:** Sections and items assessed in the Postural Assessment Scale for post-stroke individuals.

Sections	Items	Score
Maintaining a posture	1. Sitting without support	0–3
	2. Standing with support	0–3
	3. Standing without support	0–3
	4. Standing on nonparetic leg	0–3
	5. Standing on paretic leg	0–3
Changing a posture	6. Supine to paretic side lateral	0–3
	7. Supine to nonparetic side lateral	0–3
	8. Supine to sitting up on the edge of the mat	0–3
	9. Sitting on the edge of the mat to supine	0–3
	10. Sitting to standing up	0–3
	11. Standing up to sitting down	0–3
	12. Standing, picking up a pencil from the floor	0–3

**Table 2:** Characteristics of the study sample (*n* = 61).

Variable	Data
Age, years	62.75 (13.31)
NIHSS immediately after admission	13.48 (6.185)
Sex (male/female), *n*	40/21
Type of stroke (ischemic/hemorrhagic), *n*	47/14
Affected hemisphere (right/left/bilateral/subcortical), *n*	34/21/3/3
Vascular territory (basilar/middle cerebral artery/anterior cerebral artery/internal carotid artery/ anterior communicating artery/diffused), *n*	8/38/3/1/1/10

NIHSS is National Institute of Health Stroke Scale. Age and NIHSS are expressed in mean and standard deviation. Sex, type of stroke, affected hemisphere and vascular territory are frequencies.

**Table 3:** Scoring in acute, subacute and chronic phase.

	Acute Phase *n* = 61	Subacute Phase *n* = 61	Chronic Phase 1 *n* = 58	Chronic Phase 2 (One Year) *n* = 42
PASS	4 ± 16	18 ± 22	25 ± 22	30 ± 20
BI	5 ± 25	45 ± 55	60 ± 73	75 ± 58
FIM	42 ± 35	80 ± 48	91 ± 56	103 ± 50
FAC	0 ± 0	1 ± 4	3 ± 4	4 ± 4
WGS	42 ± 0	42 ± 16.60	28.65 ± 23.65	22 ± 24.90

Data are expressed in mean and standard deviation.

**Table 4:** Construct validity of postural assessment scale for stroke patients.

	First Evaluation (Acute Phase/Admission) *n* = 61			Second Evaluation (3 Months) *n* = 61			Third Evaluation (6 Months) *n* = 58			Fourth Evaluation (One Year) *n* = 42		
Tools	*r*	CI95%	*p*	*r*	CI95%	*p*	*r*	CI95%	*p*	*r*	CI95%	*p*
BI	0.801	0.68 to 0.87	<0.01	0.888	0.82 to 0.93	<0.01	0.894	0.83 to 0.93	<0.01	0.844	0.75 to 0.90	<0.01
FIM	0.678	0.51 to 0.79	<0.01	0.810	0.70 to 0.88	<0.01	0.824	0.72 to 0.89	<0.01	0.837	0.74 to 0.9	<0.01
FAC	0.791	0.67 to 0.87	<0.01	0.897	0.83 to 0.94	<0.01	0.863	0.78 to 0.91	<0.01	0.893	0.83 to 0.93	<0.01
WGS	−0.646	−0.77 to −0.47	<0.01	−0.847	−0.9 to −0.75	<0.01	−0.892	−0.93 to −0.82	<0.01	−0.890	−0.93 to −0.82	<0.01

*r*; Critical level of *p* < 0.05 was considered significant. CI 95% (95% confidence interval).

## Data Availability

Not applicable.
